# A Rare Combination of Ovarian and Uterine Leiomyomas with Goblet Cell Carcinoid of the Appendix

**DOI:** 10.1155/2015/467243

**Published:** 2015-01-19

**Authors:** Abdulrahman F. Al-Shaikh, Abdulla Darwish, Veena Nagaraj, Abeer Alsada

**Affiliations:** ^1^Department of Surgery, Bahrain Defence Force Hospital, P.O. Box 28743, Riffa, Bahrain; ^2^Department of Pathology, Bahrain Defence Force Hospital, P.O. Box 28743, Riffa, Bahrain; ^3^Department of OBGYN, Bahrain Defence Force Hospital, P.O. Box 28743, Riffa, Bahrain

## Abstract

We present a case of the rare combination of unilateral ovarian leiomyoma, uterine leiomyoma, and goblet cell carcinoid tumor of the appendix in a premenopausal woman who presented with right iliac pain. Immunohistochemistry study for desmin (muscle marker) and chromogranin and synaptophysin (neuroendocrine markers) confirmed immunophenotyping origin. Interestingly, both tumors showed positive reaction for estrogen receptor. To our knowledge, such a combination has not been reported previously in the literature. In this paper, the pathogenesis and differential diagnosis of both types of tumors are discussed.

## 1. Introduction

Ovarian leiomyoma is one of the rarest tumors of the ovary and accounts for 0.5 to 1% of all benign ovarian tumors [1] with approximately 60 cases reported in the literature [2, 3]. Most cases of ovarian leiomyoma are discovered accidentally, small in size and unilateral, and occur mostly in premenopausal women. On the other hand, goblet cell carcinoid is a type of neuroendocrine tumor that arises in the appendix and is known to exhibit aggressive behavior. The management of such malignant tumors requires an aggressive approach and regular follow-up.

Both carcinoid tumor and ovarian leiomyoma are included in the differential diagnosis of right abdominal pain. In this paper, we are reporting a rare combination of ovarian leiomyoma, uterine leiomyoma, and goblet cell carcinoid of the appendix in a 52-year-old premenopausal woman; to our knowledge, this association has not been reported in the medical literature.

## 2. Case Presentation

A 52-year-old premenopausal female presented at the emergency department with acute right-lower abdominal pain for one-month duration that was aggravated in the four days before admission. Examination of the abdomen revealed right iliac fossa tenderness, associated with rebound and guarding. The clinical findings were consistent with acute appendicitis. One year earlier, she had a full colonoscopy that showed a single colonic polyp, diagnosed microscopically as tubular adenoma. Four years ago, she had antral gastric biopsy which revealed mild chronic antral gastritis.

During her admission, an ultrasound of the abdomen and pelvis was performed and showed retrocecal appendix with early signs of inflammation (edematous wall) and a 9 × 4.6 cm uterus with a small hypoechoic fundal interstitial fibroid. A 2.5 × 2.9 cm right adnexal solid mass was also seen, with no follicles within and good vascularity. It showed intimate relation to both the right lateral uterine wall and the right ovary. A differential diagnosis of either a subserosal fibroid or a complicated ovarian cyst was suggested.

**Figure 1:**
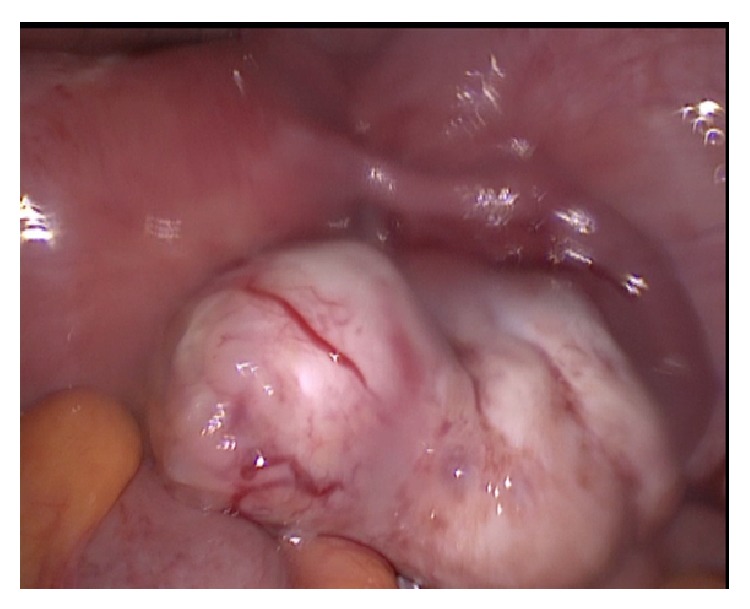
Laparoscopic intraoperative view of the right ovary, right tube, and part of the uterus. The right ovary is noted to be enlarged with a 5 × 3 cm hard mass separated from the uterus not adherent to or infiltrating the surroundings.

However, she was urgently taken to the operating room for emergent laparoscopic appendectomy. On exploring the abdominal cavity, minimal fluid was found in the peritoneum. The right ovary was noted to have a 5 × 3 cm hard mass, separated from the uterus, not adherent to or infiltrating the surroundings (Figure 1). The left ovary was within normal size, showing 2 small cystic lesions. The appendix showed congestion and hyperemia. Appendectomy was completed by the surgical team and then right salpingo-oophorectomy was done by an OBGYN team. Both specimens were sent for pathological examination.

**Figure 2:**
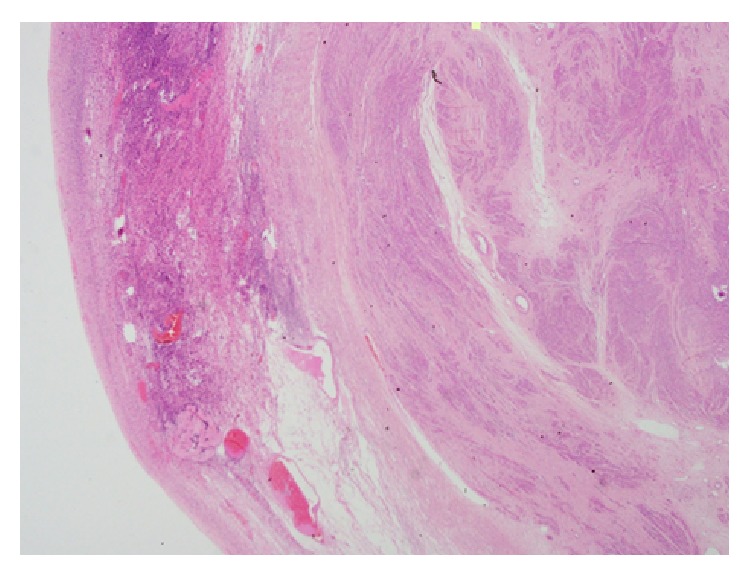
Low power microscopic view showing a normal ovarian tissue in the left side with a well-defined leiomyoma in the right side of the picture (H&E).

**Figure 3:**
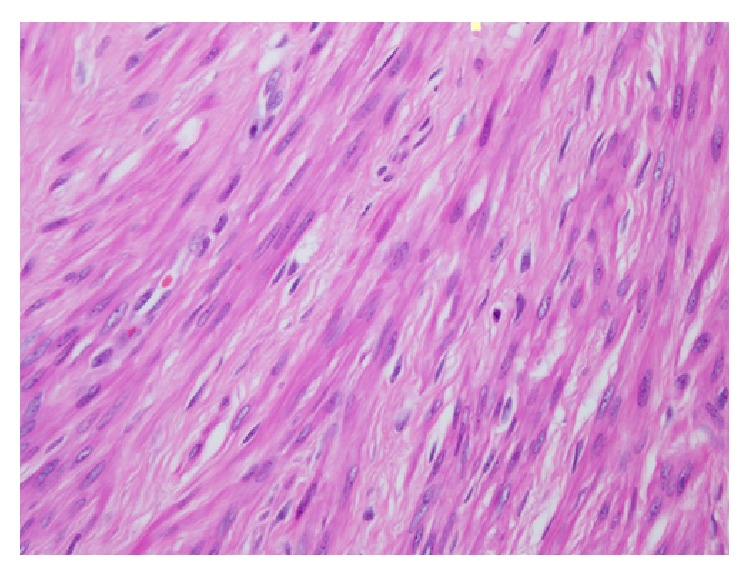
High power view of the ovarian leiomyoma composed of interlacing spindle cells with characteristic cigar-shaped nuclei (H&E).

**Figure 4:**
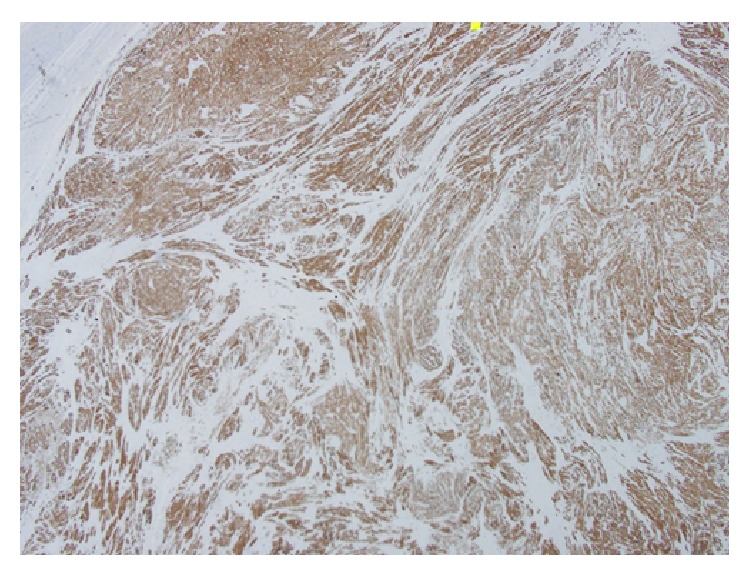
Low microscopic view shows diffuse strong positivity of the ovarian leiomyoma cells for desmin marker confirming smooth cell origin (immunohistochemistry stain).

Grossly, a 5 × 2.5 × 2 cm right ovary with attached 5 × 1 × 0.7 cm fallopian tube was received. The cut surface of the right ovary was solid and white with whorled appearance. Microscopically, it showed a normal ovarian tissue with spindle cell tumor. The tumor cells were arranged in interlacing bundles with eosinophilic cytoplasm and cigar-shaped nuclei; no significant mitosis or pleomorphism was seen (Figures 2 and 3). The tumor cells showed diffuse strong positive for desmin reaction and negative reaction for S100, inhibin, and CD68 markers, confirming the diagnosis of leiomyoma of the ovary (Figure 4).

**Figure 5:**
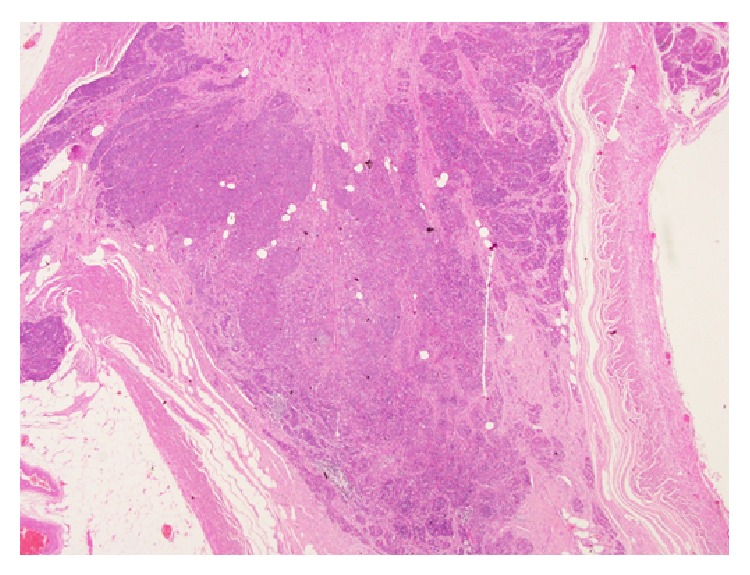
Low power microscopic view of the goblet cell carcinoid involving the entire wall to serosal level of the distal part of the appendix (H&E stained).

**Figure 6:**
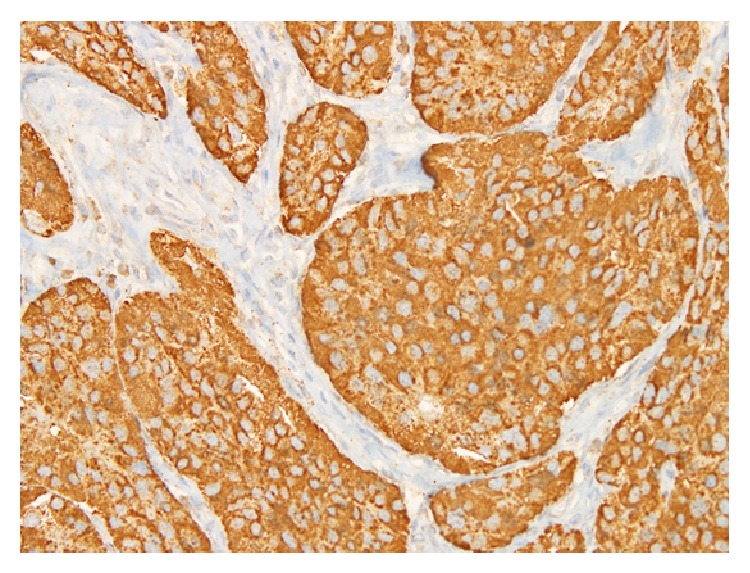
Microscopic view of the goblet cell carcinoid showing strong positive reaction of the tumor for the chromogranin marker (immunohistochemistry stain).

Additionally, a 6.5 × 1.5 × 1 cm specimen of lusterless appendix was received for histopathological examination. Microscopically, the appendix showed a tumor in the distal portion, measuring 8 mm in maximum dimension (Figure 5). The tumor cells were polygonal with eosinophilic granular cytoplasm and central, round to oval, and moderately pleomorphic nuclei having a salt-and-pepper chromatin pattern. The tumor cells were arranged in nests with focal acinar patterns separated by thin fibrocollagenous stroma. They showed focal goblet cell differentiation with positive for mucin stain and amounted to 5–10% of the total tumor volume. The tumor was present in the submucosa and the muscularis propria extending to the subserosa. There was focal vascular invasion. The tumor cells showed strong positivity for synaptophysin and chromogranin (Figure 6), confirming the diagnosis of goblet cell carcinoid tumor. Interestingly, estrogen receptor marking was performed in both tumors and showed strong positive reaction in the cells of each.

## 3. Discussion

Leiomyoma arising in the ovary is considered a rare type of benign tumor compared to other leiomyomas in the female genital tract. The size of ovarian leiomyoma is usually very small—millimeters or a few centimeters in diameter. In our case the tumor measured 5 cm in maximum dimension and was easily visible on ultrasound. Malignant potential is suspected if the tumor is huge, despite its benign microscopic picture [4]. Most of these tumors are unilateral. Interestingly, most of the bilateral cases are diagnosed in young patients [5]. The oldest age reported in a bilateral case is 37 years [6].

Usually the presentation of ovarian leiomyoma occurs in the premenopausal, childbearing years. This is also the common age for developing uterine leiomyoma. However, postmenopausal patients represent approximately 16% of cases [2, 7]. There is association of ovarian leiomyoma and intrauterine leiomyoma in our case, and this may help us to understand the mechanisms of development of such tumors.

Ovarian leiomyomas are mainly asymptomatic and are discovered accidentally during imaging or operation for uterine leiomyoma or other pathologies [8]. The patient in our case presented with a one-month history of right iliac fossa pain and then developed symptoms and signs of acute appendicitis. The presentation of a carcinoid tumor tends to be asymptomatic or accidentally discovered, or with symptoms of acute appendicitis [9–11]. We suppose that the month of abdominal pain in our patient was most likely due to the ovarian leiomyoma, but the aggravated, acute abdominal pain was most probably due to acute appendicitis related to the carcinoid tumor.

Leiomyoma of the ovary may not always present asymptomatically or with abdominal pain. A rare presentation was reported by Kurai et al. [3] who reported leiomyoma of the ovary presented with Meigs syndrome, which disappeared after removal of the ovary. Other rare presentations have included lower abdominal mass [12], ascites with hydrothorax [13], ascites with polymyositis [14], ascites with elevated CA125 [15], or even hydronephrosis as a consequence of its huge size [16].

The histogenesis of ovarian leiomyoma is not well known. Some theories hypothesize that the tumor may originate from hilar blood vessels, smooth muscle metaplasia of ovarian stroma, or smooth muscle-like theca externa cells [8]. Its association with uterine leiomyoma may suggest that they share the same mechanisms of development. This theory is explained by the rapid growth of such tumors during pregnancy and their positivity for estrogen and/or progesterone receptors [8]. Tomas et al. have suggested that ovarian leiomyoma could arise from smooth muscle metaplasia of endometriotic stroma, or it could be derived from myofibroblasts that originate from metaplastic ovarian stromal cells present in the rim of the endometriotic cyst especially if the tumor was associated with endometriosis or endometriotic cysts [1]. In our case, there is no evidence of endometriosis, but the tumor is associated with fundal uterine fibroid.

The presence of normal ovarian tissue beside the tumor confirms the ovarian origin of the tumor and excludes tumors of other origins, such as leiomyoma of broad ligament or a subserous leiomyoma that grew large and lost its attachments to the uterus (wandering leiomyoma) [17]. Despite its rarity, leiomyosarcoma, which has a characteristic microscopic appearance, should also be considered in the differential diagnosis.

Apart from leiomyoma there are other ovarian tumors that show a spindle cell microscopic appearance. Fibroma is the most common ovarian spindle cell neoplasm, but other neoplasms of the sex cord-stromal group may contain spindle cells and the differential diagnoses may include thecoma, granulosa cell tumor, Sertoli-Leydig cell tumor, sclerosing stromal tumor, and signet-ring stromal tumor [18]. However, immunohistochemistry testing for desmin, a marker for smooth muscle cells, is in difficult cases. It shows strong diffuse positivity in our case. Other markers for smooth muscles are *α*-smooth muscle actin and h-caldesmon. Sometimes it may be difficult to differentiate between ovarian leiomyoma and ovarian fibroma, but Lerwill et al. [8] concluded in their study that smooth muscle tumors show diffuse desmin reaction while fibromatous tumors show either negativity or focal positivity with desmin. Also, smooth muscle actin is often positive in fibromatous tumors, so it is not useful to differentiate between the two tumors [8].

On the other hand, goblet cell carcinoid of the appendix was first described as a separate entity in 1974 [19, 20]. In general, goblet cell carcinoid is intermediate between adenocarcinoma and carcinoid tumor in terms of age at presentation, likelihood spread to other organs or lymph node involvement, and outcome [21]. However, when it comes to treatment options, goblet cell carcinoid is often treated aggressively as adenocarcinoma rather than carcinoid tumor [22]. In our case, only an appendectomy was done, with regular clinical and CT follow-up, with no evidence of residual tumor.

To our knowledge there is no case report describing an association between goblet cell carcinoid tumor of the appendix and leiomyoma of the ovary or leiomyomas in general. A single case of carcinoid tumor of the appendix was reported in a patient with endometrial cancer and uterine myoma [23].

## 4. Conclusion

The association of a rare goblet cell carcinoid tumor of the appendix with ovarian leiomyoma has not been reported in the literature. Both tumors are of uncertain histogenesis, but the shared presence of estrogen receptors may indicate that both tumors are hormone dependent. Despite its rarity, ovarian leiomyoma should be considered in the differential diagnosis of acute abdomen and other spindle cell tumors of the ovary. Extensive sampling, along with appropriate immunohistochemistry studies, should be performed to confirm diagnosis.
